# Optimizing photon counting detector CT reconstructions for automatic vessel tracing in prostate artery embolization

**DOI:** 10.1002/acm2.70835

**Published:** 2026-10-06

**Authors:** Taylor Froelich, Forrest B. Linch, Scott M. Thompson, Michael C. Jundt, Jeremy D. Collins, Andrea Ferrero, Christopher P. Favazza

**Affiliations:** ^1^ Department of Radiology Mayo Clinic Rochester Minnesota USA

**Keywords:** photon‐counting‐detector CT (PCD‐CT), procedural guidance, prostate artery embolization (PAE), workflow improvement

## Abstract

**Background:**

Prostate artery embolization (PAE) requires precise visualization of complex pelvic arterial anatomy and can be aided by advanced vessel navigation tools. Photon counting detector (PCD) CT provides simultaneous ultra‐high resolution and material‐specific imaging, potentially improving automatic vessel tracing workflow for procedural planning and guidance.

**Purpose:**

To identify, within a PCD‐CT workflow, the reconstruction that provides the most robust and consistent automatic vessel tracing for pre‐procedural PAE planning.

**Methods:**

Ten patients underwent PCD‐CT angiography of the prostate arteries in ultra‐high‐resolution mode. Four reconstruction approaches were evaluated: conventional reconstructions with (1) standard of care kernel and (2) sharper kernel (Bv64), along with (3) monoenergetic 50 keV, and (4) iodine map spectral results. Using commercial embolization guidance software, three vessels were manually traced for each patient to establish ground truth segmentation. Subsequently, automatic vessel traces were generated using identical start/end points as the ground truth. Root mean squared error (RMSE) between automatic traces and ground truth was used to quantify tracing accuracy.

**Results:**

Comparing individual traces, iodine maps demonstrated consistency with median RMSE of 0.7 mm (0.4 mm interquartile range (IQR)) versus 0.7 mm (12.5 mm IQR), 1.5 mm (14.7 mm IQR), and 0.8 mm (6.7 mm IQR) for Bv56, Bv64, and 50 keV reconstructions, respectively. Iodine maps produced fewer outliers, with 13.3% of traces and 40.0% of patients having one or more traces exceeding 5 mm RMSE, compared with 26.7%–36.7% of traces and 60.0%–80.0% of patients for the other reconstructions.

**Conclusions:**

Material‐specific iodine reconstructions from PCD‐CT yield more robust automatic vessel tracing for PAE planning, offering a workflow‐compatible solution requiring minimal protocol modifications.

## 1 INTRODUCTION

Prostate artery embolization (PAE) has emerged as a minimally invasive treatment for benign prostatic hyperplasia and requires precise identification of prostatic arterial anatomy for procedural success.^1^, ^2^ Each patient's unique pelvic arterial anatomy presents clinical challenges in patient selection and procedural planning. Severe atherosclerotic disease may preclude PAE entirely, and knowledge of tortuous pelvic arterial anatomy and/or acutely angled prostatic artery origins and numbers can inform arterial access site selection, required supplies, and available sedation resources. Further, detection of potential intra‐ or extra‐prostatic collateral vessels may modify treatment approach, as they can be a source of non‐target embolization. Challenges may also arise during PAE, whereby intra‐procedural navigation of complex arterial anatomy, including identification and selection of the desired prostatic artery(ies), can pose diagnostic, and by extension therapeutic, dilemmas.^3^, ^4^

To address the aforementioned clinical challenges of PAE, an approach utilizing pre‐procedural prostate artery CT Angiography (CTA) integrated with commercial embolization guidance software can be employed for vessel mapping, procedural planning, and intraprocedural navigation. This workflow potentially improves procedural efficiency and outcomes by enabling detailed pre‐procedural vessel analysis and real‐time procedural guidance, highlighting vessels that may not opacify well on conventional angiograms.

Photon counting detector (PCD)‐CT represents a fundamental advancement from conventional energy‐integrating detectors, directly converting X‐ray photons into electrical signals while preserving spectral information.^5^, ^6^, ^7^ This technology enables simultaneous ultra‐high spatial resolution imaging and material‐specific reconstructions, providing enhanced visualization of small arteries. While material‐specific reconstructions, such as iodine maps and virtual monoenergetic images, can also obtained using standard dual‐energy EID‐CT, the unique combination of ultra‐high spatial resolution and spectral data has led to the adoption of PCD‐CTA as the default imaging modality for pre‐procedural PAE evaluation at our institution.^8^

While PCD‐CT image data have been successfully integrated with embolization guidance software in clinical practice, current implementations face limitations.^8^ In many instances, the software does not properly trace vessels and requires prolonged processing times. To date, PCD‐CT image reconstructions have not been optimized specifically for vessel tracing and procedural guidance, highlighting the need to optimize reconstruction selection within this workflow to maximize the robustness of automatic vessel tracing applications.

This technical note evaluates several PCD‐CT reconstruction approaches to identify which provides the most robust and consistent automatic vessel tracing to streamline PAE procedural planning and guidance.

## 2 METHODS

This retrospective study, conducted with Institutional Review Board approval, included 10 patients who underwent clinically indicated pelvic PCD‐CTA for PAE planning in January and February 2025. All examinations were performed on a Siemens NAEOTOM Alpha PCD‐CT scanner utilizing an institution‐developed pre‐PAE exam protocol: 125 mL iodinated contrast at 6 mL/s, with image acquisition triggered 12 s after reaching 150 HU threshold in the descending aorta. Ultra‐high‐resolution (UHR) mode was employed for image acquisition along with the following parameters: 120 kV, 165 IQ level, 120 × 0.2 mm collimation, 0.25 s rotation time and 0.6 helical pitch. For distal branch opacification, 0.8 mg nitroglycerin was administered sublingual 5 min prior to scanning.

For all patients, images were reconstructed from near the iliac crest through the lesser trochanter using a 250 mm field of view (FOV) centered over the bladder. Four reconstruction approaches were evaluated utilizing the thinnest slice thickness available, including (1) Standard: T_low_ data (20‐120 keV), Bv56 kernel, 0.2 mm slices; (2) Sharp kernel: T_low_ data, Bv64 kernel, 0.2 mm slices; 3) 50 keV virtual monoenergetic, Bv56 kernel, 0.4 mm slices; and (4) Iodine maps, Bv56 kernel, 0.4 mm slices. All reconstructions utilized a 0.2 mm increment. In Figure 1, an axial slice of the 4 reconstructions is shown for a representative patient in the cohort.

**FIGURE 1:**
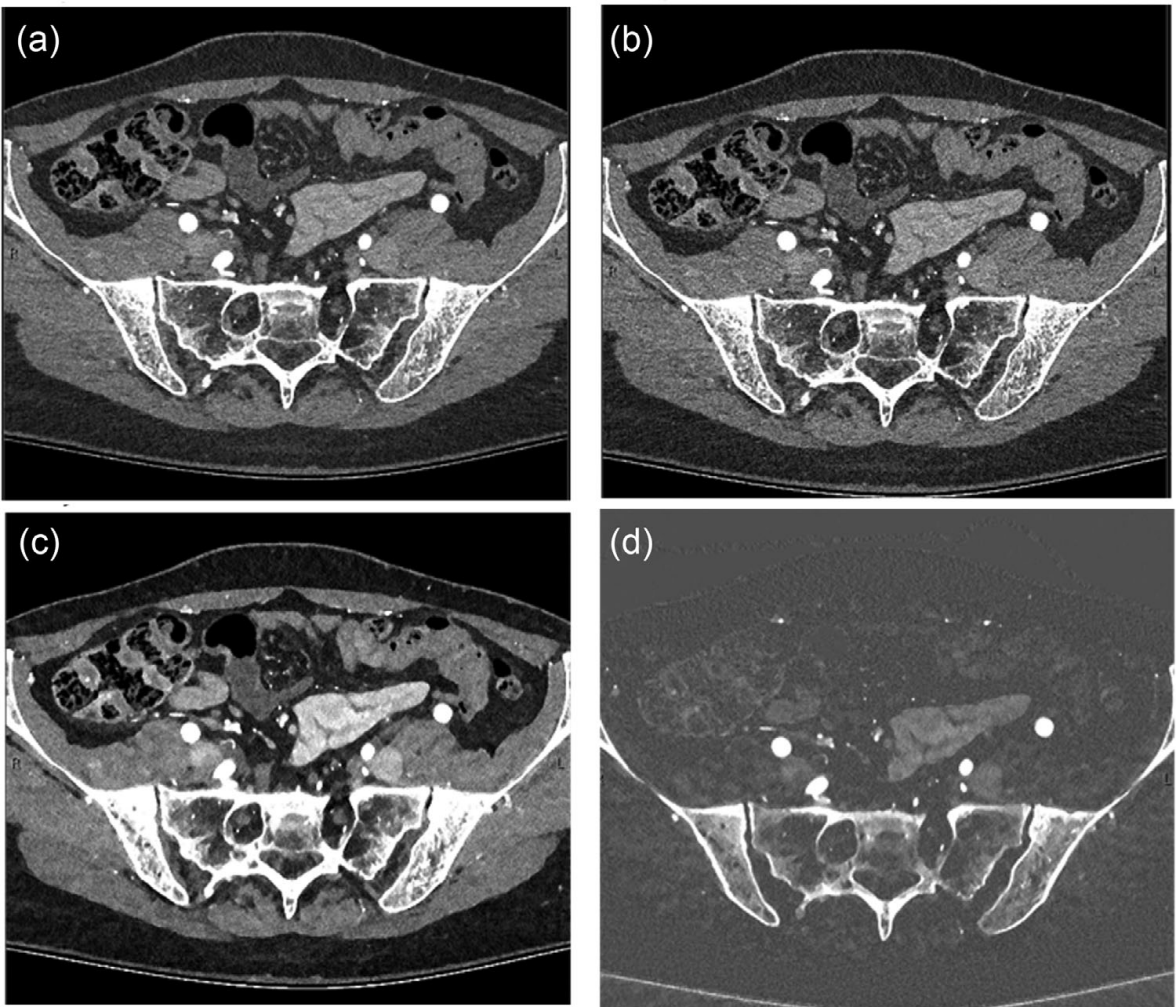
PCD‐CT reconstruction approaches evaluated for vessel tracing accuracy. Four reconstruction methods were compared: (a) Standard clinical reconstruction (T_low_ data, Bv56 kernel, 0.2/0,2 mm slice thickness/increment), (b) Sharp kernel reconstruction (T_low_ data, Bv64 kernel, 0.2/0.2 mm slice thickness/increment), (c) 50 keV virtual monoenergetic images (Bv56 kernel, 0.4/0.2 mm slice/increment), and (d) Iodine maps (Bv56 kernel, 0.4/0.2 mm slice thickness/increment). All images show the same axial slice for direct comparison of vessel conspicuity and image quality across reconstruction techniques.

Using Siemens Syngo Embolization Guidance software and under the guidance of a board‐certified interventional radiologist, a clinical medical physics resident identified and manually traced three arterial vessels per patient on the standard reconstruction to establish ground truth, with seed points placed every 3–5 slices along the entire vessel course. Automatic vessel traces were subsequently generated for each reconstruction using the same software. To ensure consistency, automatic tracing utilizes identical start and end points as the ground truth traces. In Figure 2, the automated traces are shown superimposed to the ground truth for a representative vessel.

**FIGURE 2:**
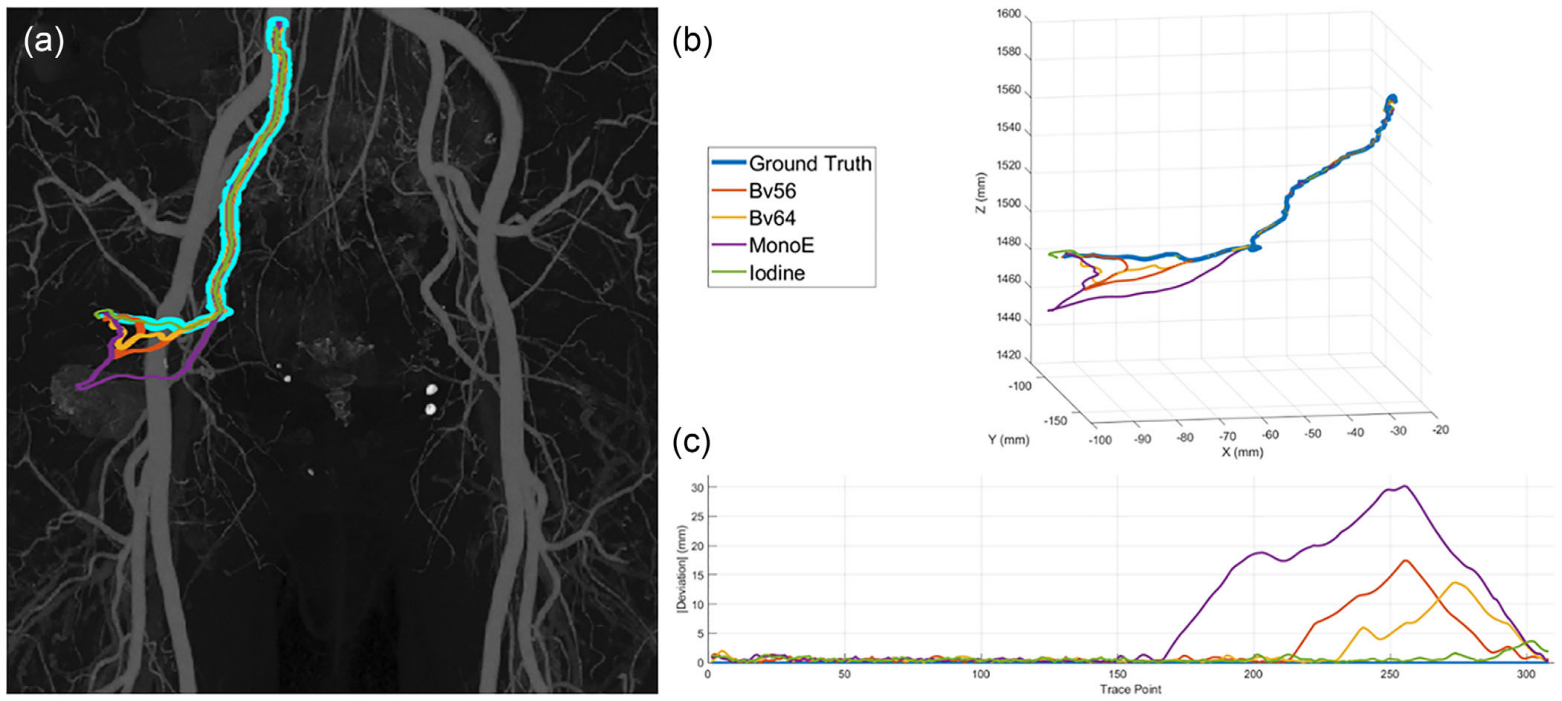
Assessment of automatic vessel tracing accuracy against manual ground truth. (a) Coronal maximum intensity projection (MIP) image showing manual ground truth trace (cyan) and automatic traces from different PCD‐CT reconstructions (Bv56, Bv64, VMI, Iodine) for a representative vessel in one patient. The widths of the tracings vary for visualization purposes only. (b) 3D visualization of traces demonstrating spatial deviations, with larger errors in tortuous vessel regions. (c) Absolute deviation from ground truth versus trace point, showing progressive increase toward the distal vessel end. Minimum distances between automatic and ground truth trace points were measured, and RMSE was calculated for accuracy assessment.

Trace accuracy was quantified by calculating the minimum Euclidean distance between each point on the automatic trace and the nearest point on the ground truth trajectory. Root mean squared error (RMSE) was computed for each trace:

$$\;RMSE_{trace}=\sqrt{\frac{1}{n}\sum_{i=1}^{n}d_{i}^{2}}$$

where $d_{i}$ represents the minimum distance from the $i^{th}$ automatic trace point to the closest point in the ground truth trace and n is the total number of trace points. Given the non‐normal distribution of errors skewed by trace failures, median and interquartile range (IQR) were used as primary descriptive statistics. To investigate the consistency of performance, a bimodality assessment was conducted by categorizing traces as “successful” (RMSE ≤ 5 mm) or “failed” (RMSE > 5 mm); this threshold was found to best capture the interventional radiologist's clinical assessment of the trace overlays.

## 3 RESULTS

Evaluating the 30 vessel tracings across all patients individually (Figure 3), all 4 reconstructions yielded similar median RMSE values—0.7–1.5 mm. However, there were greater differences in IQR across the different reconstruction with the lowest IQR generated by iodine maps (0.4 mm), followed by 50 keV images (6.7 mm), Bv56 (12.5 mm) and lastly, Bv64 (14.7 mm). The greater consistency in performance with iodine maps is also demonstrated through the binary tracing pass/fail analysis. Using a 5 mm RMSE as the classification threshold, iodine maps produced only 4 of 30 failed traces as compared to 10 of 30 for Bv56, 11 of 30 for Bv64, and 8 of 30 for monoenergetic images.

**FIGURE 3:**
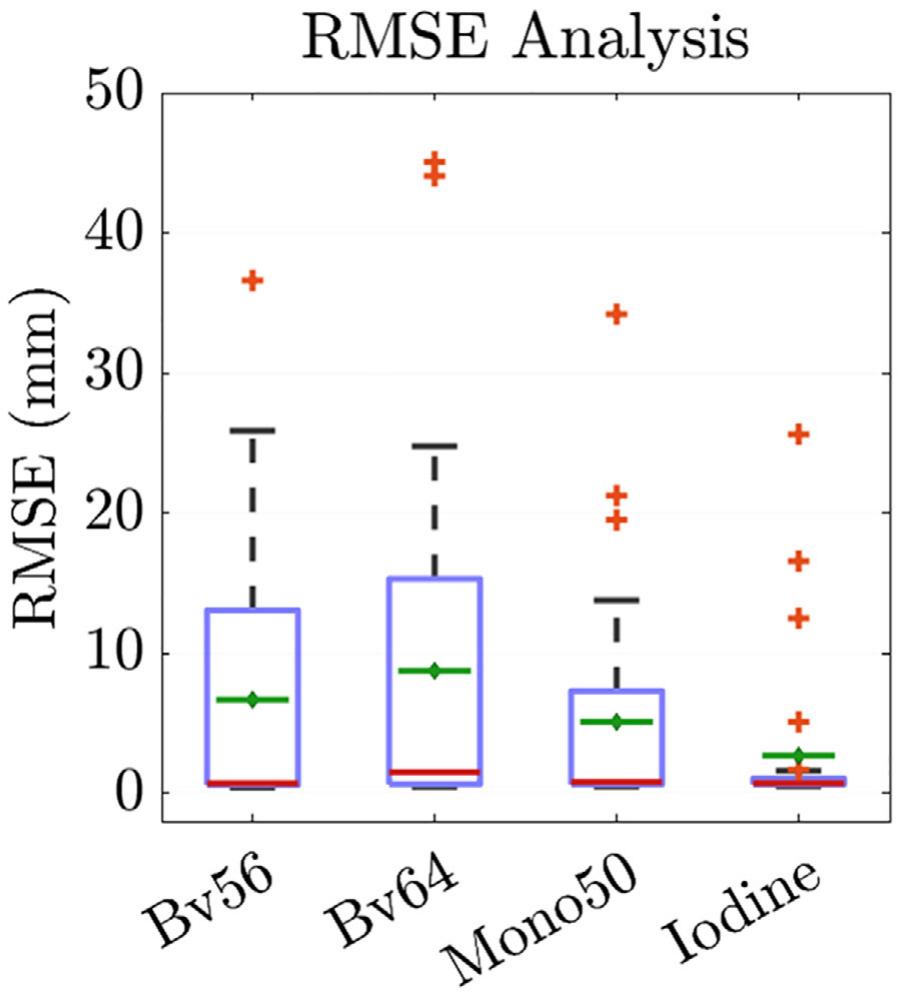
RMSE comparison across reconstruction methods for vessel tracing. Trace‐level analysis for all individual vessel traces (*n* = 30 traces from 3 vessels × 10 patients). The box plot displays the median (red line), interquartile range (blue box), whiskers extending to 1.5 × IQR, and outliers (orange‐red circles). Green diamonds and horizontal lines represent group means. Iodine maps achieved a median RMSE of 0.7 mm (IQR: 0.4 mm), with 13.3% of traces exceeding the 5 mm threshold, compared with 26.7%–36.7% for the other reconstructions.

In Figure 4, the paired trace analysis compares the accuracy of each alternative reconstruction directly against the standard Bv56 reconstruction. Data points for the iodine map reconstruction (blue markers) consistently fall below the unity diagonal line representing equal performance with the reference trace, with only one trace (arrow) where it resulted in a substantially higher RMSE compared to the reference protocol. Conversely, multiple traces with low RMSE on the reference protocol resulted in high RMSE on the other two alternative reconstructions, and vice versa.

**FIGURE 4:**
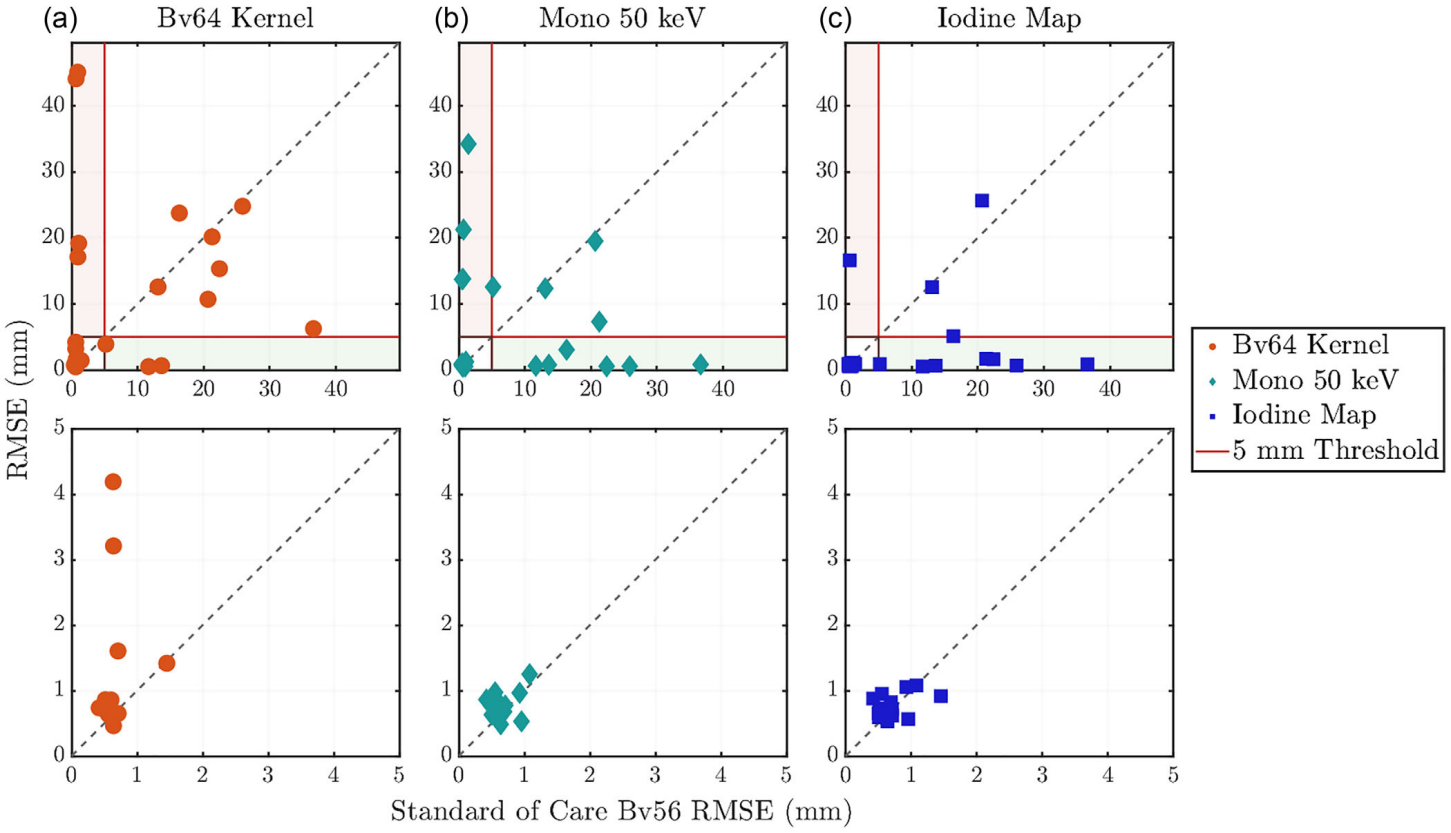
Paired trace analysis comparing the RMSE of the clinical standard Bv56 reconstruction (x‐axis) against the three alternative methods on the y‐axis: (a) Bv64, (b) monoenergetic 50 keV, and (c) iodine maps. The top row displays the full RMSE range (0–50 mm), while the bottom row provides a zoomed‐in view of the 0 to 5 mm region corresponding to the 5‐mm threshold. The dashed diagonal line (y = x) represents equal performance. Points falling in the lower‐right region (below the diagonal) indicate cases where the alternative method demonstrated a lower RMSE compared to the standard.

Patient‐level analysis (Figure 5) assessed the distribution of these larger deviations by quantifying how many of the three vessel traces exceeded the 5 mm RMSE threshold for each of the 10 patients. Generally, failed vessel tracings were distributed across patients for each of the different image reconstructions. For iodine maps, four failures occurred in four different patients. For the monoenergetic 50 keV images, the 8 failed traces occurred in 6 patient exams. The ten vessel tracing failures in Bv56 images were spread across 8 patients and the 11 failures from Bv64 were found in 7 different patients.

**FIGURE 5:**
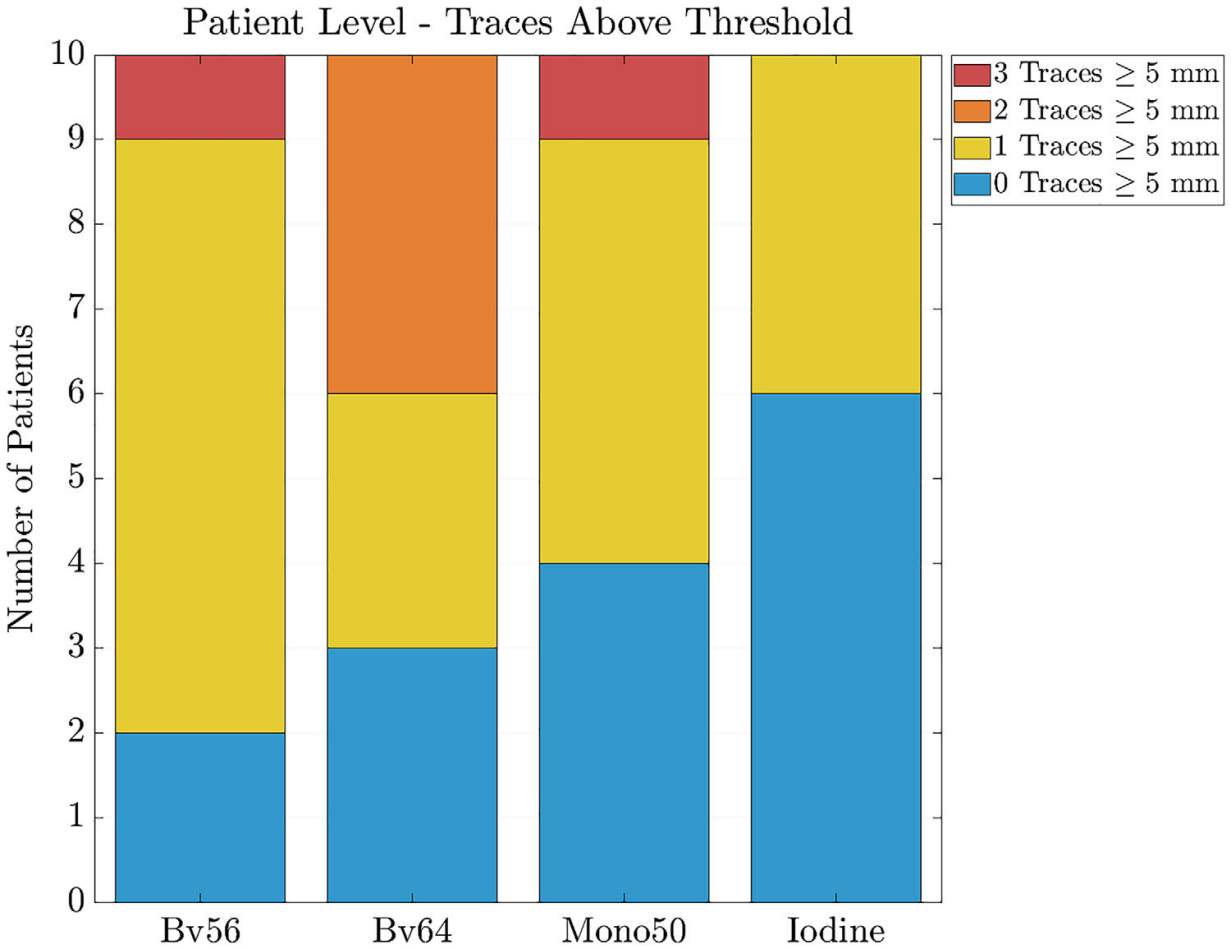
Patient‐level distribution of traces exceeding the 5 mm RMSE threshold (*n* = 10 patients). The stacked bar chart illustrates the number of patients with 0, 1, 2, or 3 traces exceeding the threshold for each reconstruction. Iodine maps demonstrated the greatest reliability, with all patients having one or fewer traces above the threshold (60% with zero, 40% with one). The Bv64 kernel produced the highest frequency of patients with multiple failed traces (40% with two). For conventional Bv56 and monoenergetic 50 keV, most failures occurred as a single trace per patient (70% and 50% of patients, respectively), with 10% of patients in each group having all three traces exceed the threshold.

## 4 DISCUSSION

Across 30 vessel traces (3 vessels × 10 patients), iodine map reconstructions yielded the most consistently accurate traces compared to all other reconstructions. Although multiple reconstructions yielded a sub‐millimeter median RMSE values, iodine maps yielded a much narrower IQR—0.4 mm versus 6.7–14.7 mm for the other reconstructions. There are numerous patient‐specific sources of variability that can affect CTA exams, including patient size, cardiac output, iodine contrast injection rate and amount, and so forth. Consequently, these differences could affect the performance of the vendor's automatic tracing tool. Patient exams for this study were chosen without selection criteria and represent a small but random sampling of the patient population. For this small sample of patients, the instances in which the tracing software failed to accurately identify the vessel path were distributed across patients and was not a dominant factor. These results suggest that the improved reliability in performance with the iodine maps generalize across patients.

The improved performance of the iodine maps stems in part from the vendor's specific approach to automated vessel tracing. The software utilizes a cross‐sectional, model‐based minimum‐cost‐path algorithm that extracts the centerlines between two user‐defined start and end seed points.^9^ Material decomposition isolates the iodine signal from background tissues, maximizing vessel‐to‐background contrast. This enhanced conspicuity facilitates more reliable vessel boundary detection by the automatic tracing algorithm. Additionally, iodine maps inherently minimize the relative contribution of calcification and bone, reducing potential confounders that could misdirect vessel traces. As a result, the material‐specific nature of iodine maps provides more consistent signal intensity along vessel paths, potentially reducing discontinuities that can terminate automatic traces prematurely.

Conversely, while sharper kernels with ultra‐thin (0.2 mm) slices improved vessel edge definition compared to standard reconstructions, this enhancement did not translate to improved automatic tracing accuracy. The degraded performance of Bv64 compared to standard Bv56 reconstruction (higher IQR and number of failed traces) suggests that increased kernel sharpness may introduce noise or artifacts that adversely affect automated vessel detection algorithms.

Enhanced pre‐procedural planning accuracy may reduce procedure time by minimizing the need for exploratory angiography and vessel searching during PAE. Clinical experience has demonstrated that the embolization guidance overlay can be used to identify optimal working angles without performing additional digital subtraction angiography. More accurate vessel mapping could also improve target vessel selection and reduce non‐target embolization risk.

A key strength of this approach is its seamless integration with existing clinical workflows. The iodine map reconstruction can be generated automatically as part of the standard PCD‐CT protocol, requiring no additional scan time or radiation exposure. The reconstructed images are directly compatible with commercial embolization guidance software, eliminating the need for specialized post‐processing.

Several limitations warrant consideration. First, trace accuracy was assessed using manually defined ground truth, which may introduce observer‐dependent variability. Second, the study focused solely on trace accuracy and did not evaluate clinical outcomes such as procedure time or embolization success rates. Third, while traces were selected to be representative of clinical practice in terms of vessel length, size and tortuosity, their starting and ending point has an effect on the reported RMSE metric and could affect the comparison across different traces.

The embolization guidance software used in this investigation was likely designed to ingest lower density image data (i.e., conventional CT or MRI). PCD‐CT images with large matrix sizes and thin slices, covering the standard clinical reconstruction range, could not be fully loaded into the software due to computer memory constraints; partial series could be loaded only. This limitation introduces workflow complications and requires the user to manually select a subset of images. For all image series‐, the craniocaudal coverage was reduced from the standard clinical reconstruction range to focus on the vessels of interest only, thus allowing the entire image series to be loaded. For all reconstructions, each automatic trace used the same start and end points as its ground truth and the full vessel was contained within the contiguous loaded range. For the standard reconstruction range prescribed by the clinical protocol, the entire series generated with a 768 × 768 matrix and 0.4/0.3 slice thickness/increment could be fully loaded into the application without penalty. For the small (250 mm) FOV reconstruction in the exam protocol, a 768 × 768 matrix is sufficiently large to capture the full spatial resolution of the reconstruction and 0.4 mm is the thinnest slice thickness for spectral results.

## 5 CONCLUSIONS

In this preliminary study, material‐specific iodine reconstructions from PCD‐CT provided the most robust and consistent automatic vessel tracing among the reconstruction approaches evaluated for PAE planning. Compared with conventional reconstructions, iodine maps produced fewer traces exceeding the 5 mm threshold and a narrower error distribution, indicating more reliable automatic vessel delineation. This improvement can be achieved through a simple modification to standard reconstruction protocols without requiring changes to acquisition parameters or additional radiation exposure. These findings identify iodine‐map reconstruction as the preferred input for automatic vessel tracing within a PCD‐CT PAE workflow and motivate larger studies to confirm the benefit and to assess its impact on procedural efficiency and clinical outcomes.

## AUTHOR CONTRIBUTIONS

**Taylor Froelich**: Conceptualization; methodology; formal analysis; writing—original draft. **Forrest B. Linch**: Clinical expertise; resources; supervision. **Scott M. Thompson**: Clinical expertise; resources; supervision. **Michael C. Jundt**: Clinical expertise; resources; supervision. **Jeremy D. Collins**: Clinical expertise; resources; supervision. **Andrea Ferrero**: Conceptualization; methodology; writing—review and editing. **Christopher P. Favazza**: Conceptualization; methodology; writing—review and editing.

## FUNDING INFORMATION

This research was partially funded by Siemens Healthineers through in‐kind support (PI: Dr. Forrest B. Linch).

## CONFLICT OF INTEREST STATEMENT

The authors maintained full control of the study design, data collection, analysis and interpretation, as well as manuscript writing.

## ETHICAL APPROVAL

This retrospective quality improvement study was granted Exempt/Minimal Risk status by the Mayo Clinic Rochester Institutional Review Board. All research was conducted in strict accordance with IRB regulations and institutional ethical guidelines.

## Data Availability

Authors will share data upon request to the corresponding author.
